# Chagas Disease: Comparison of Therapy with Nifurtimox and Benznidazole in Indigenous Communities in Colombia

**DOI:** 10.3390/jcm13092565

**Published:** 2024-04-26

**Authors:** Simone Kann, Gustavo Concha, Hagen Frickmann, Ralf Matthias Hagen, Philipp Warnke, Ernst Molitor, Achim Hoerauf, Joy Backhaus

**Affiliations:** 1 Department of Microbiology and Hospital Hygiene, Bundeswehr Central Hospital Koblenz, 56070 Koblenz, Germany; ralfmatthiashagen@bundeswehr.org; 2 Institute of Medical Microbiology, Immunology and Parasitology (IMMIP), University Hospital Bonn, 53127 Bonn, Germany; molitor@uni-bonn.de (E.M.); achim.hoerauf@ukb.uni-bonn.de (A.H.); 3 Organization Wiwa Yugumaiun Bunkauanarrua Tayrona (OWYBT), Department Health Advocacy, Valledupar 2000001, Colombia; gustavoconcha16@gmail.com; 4 Department of Microbiology and Hospital Hygiene, Bundeswehr Hospital Hamburg, 20359 Hamburg, Germany; frickmann@bnitm.de; 5 Institute for Medical Microbiology, Virology and Hygiene, University Medicine Rostock, 18057 Rostock, Germany; philipp.warnke@med.uni-rostock.de; 6 Statistical Consulting, 97074 Wuerzburg, Germany; statistik.backhaus@gmail.com

**Keywords:** Chagas disease treatment, benznidazole, nifurtimox, indigenous, Colombia

## Abstract

**Background**: For indigenous people in Colombia, high infection rates with Chagas disease (CD) are known. **Methods**: In 2018 and 2020, nine villages were screened for CD. CD-positive patients could enter a drug observed treatment. While, in 2018, Benznidazole (BNZ) was provided as the first-line drug by the government, nifurtimox (NFX) was administered in 2020. **Results**: Of 121 individuals treated with BNZ, 79 (65%) suffered from at least one adverse event (AE). Of 115 treated with NFX, at least one AE occurred in 96 (84%) patients. In 69% of BNZ cases, the side effects did not last longer than one day; this applied to 31% of NFX cases. Excluding extreme outlier values, average duration of AEs differed highly significantly: BNZ (*M* = 0.7, *SD* = 1.4) and NFX (*M* = 1.7, *SD* = 1.5, *p* < 0.001). Using an intensity scale, AEs were highly significantly more severe for NFX (*M* = 2.1, *SD* = 0.58) compared to BZN (*M* = 1.1, *SD* = 0.38), *p* < 0.001. When analyzing the duration in relation to the intensity, the burden of AEs caused by NFX was significantly more pronounced. Dropouts (*n* = 2) due to AEs were in the NFX-group only. **Conclusions**: Side effects caused by BNZ were significantly fewer, as well as milder, shorter in duration, and more easily treatable, compared to NFX.

## 1. Introduction

Chagas disease (CD) belongs to the group of neglected tropical diseases. Among these, it is associated with the second highest burden of disease in Latin American countries [1,2]. The underlying pathogen, transmitted by Triatomines, is the protozoan parasite *Trypanosoma (T.) cruzi*. Triatomines belong to the family Reduviidae and the order Hemiptera (“true bugs”). While taking a blood meal, they defecate infected heces, which are incorporated by scratching the itching bite [3]. After infection, the patients enter an acute stage. In the majority of cases, this stage is characterized by flu-like symptoms. Although this stage is best for successful treatment, it is usually not diagnosed, as awareness and diagnostic options are lacking. A total of 30–40% of untreated cases progress into a symptomatic chronic stage, within which gastrointestinal and/or cardiac complications are the leading causes of premature death or disability after several years of disease progression. More comprehensive summaries have been described in various reviews [4,5,6,7,8,9,10,11,12,13,14,15,16,17].

For the treatment of Chagas disease (CD), only two drugs are routinely available: benznidazole (BNZ) and nifurtimox (NFX). Although several studies on both drugs have been published, their results in regard to efficacy, effectiveness, and side effects are partly non-conclusive. While, in general, many authors prefer benznidazole due to hints of a favorable side effect profile [18,19], this dogma is not shared by all. This has also resulted in a treatment regimen shift from benznidazole to nifurtimox for the Colombian indigenous population, which is assessed in the course of the study presented here. For readers with deeper interest in the various aspects of the discussion, the different points of view have been summarized in multiple reviews published previously [20,21,22,23,24,25,26,27,28,29,30,31,32,33,34,35,36,37,38,39,40,41,42,43,44,45,46,47,48,49,50].

As detailed in the aforementioned reviews, the side effects of benznidazole and nifurtimox are numerous and partly severe. Frequently reported benznidazole-associated side effects comprise gastrointestinal symptoms like nausea, vomiting, painful stomach, and loss of appetite; skin-related symptoms like exanthema and urticaria; as well as neuro-psychiatric symptoms like headache, pathological fear, and sleep disorders. Rare but severe side effects usually requiring therapy comprise fever, severe skin reactions like purpura, severe neurologic disorders like paresthesia and peripheral polyneuropathies, as well as myelosuppression manifesting as leukopenia or agranulocytosis. Nifurtimox has been primarily associated with gastrointestinal side effects, including weight loss up to anorexia; joint and muscle pain; and neuro-psychiatric disorders including agitation, disturbed sleep, and headache. Skin-related side effects and severe neurological symptoms like paresthesia are less frequently reported. Toxicity for testes, ovaries, and adrenal glands has been reported from animal experiments, and high rates of side-effect-related therapy stops have been shown.

The question of which drug should be first- and which should be second-line is controversially discussed. A lack of clinical evidence has changed treatment regiments provided by local governments repetitively, as can be observed for Colombia. Such courses of action are the subject of the study presented here. As is typical for pharmaceutical studies, a major portion of the available data on the effects and side effects of BNZ and NFX are provided by the producer companies, which may represent a conflict of interest and might be considered as a source of bias.

In the study presented here, we independently analyzed adverse events (AEs) and outcomes of both medications in a treatment-naïve population. This population consisted of individuals from an indigenous tribe called Wiwa, living in retracted areas of the Sierra Nevada de Santa Marta in the northeast of Colombia. In their territories, CD prevalence is high (in average 30–40%) [51], but treatment options are scarce [52]. CD spread is facilitated in Wiwa communities for various reasons, e.g., due to traditional housing (mud walls and palm roofs) providing ideal hiding places for the transmitting triatomes. Further, knowledge of the disease is poor, as are appropriate prevention measures among Wiwas. Surveillance programs in the region mainly serve the purpose of data collection, while the implementation of countermeasures is lacking. Another important obstacle for the indigenous population is the fact that—according to Colombian guidelines—only patients with two different positive ELISA tests for CD are eligible for therapy. Unfortunately, such ELISA assays are not available, apart from studies, in the region.

A screening for CD was performed with all required governmental ELISA tests and, in addition, a real-time PCR test for *T. cruzi* as well as a rapid diagnostic test (RDT). Positively tested patients had the option of participating in a drug observed treatment (DOT) of CD, if applicable. The treatment provided by the government was benznidazole during the first study phase in 2018. In the second study phase in 2020, nifurtimox was applied. In this non-blinded, prospective study, we directly compared the number, duration, and intensity of AEs in both treatment groups, as well as the outcomes in terms of real-time PCR positivity in a study conducted independently from the pharmaceutical industry. Regarding the chosen outcome parameter, it has to be noted that the transient reduction in blood parasitemia, measurable with real-time PCR, cannot definitely exclude the persistence of vital *Trypanosoma cruzi* within patient tissue, as has previously been shown for therapeutic attempts with posaconazole and fosravuconazole, while benznidazole has achieved a better reduction in serological titers, as was summarized recently [37]. Nevertheless, achievement of negative results in highly sensitive real-time PCR was used as a surrogate parameter for antiparasitic efficacy in the study presented herein. It should, however, be pointed out that this is not definite proof of a clinical cure.

## 2. Materials and Methods

### 2.1. Study Design and Study Population

The study in 2018 was called “Program against Chagas disease in the indigenous population of Colombia”. Within this program, basically all inhabitants of four indigenous villages were screened for CD. The villages were located in the Sierra Nevada de Santa Marta in the northeast of Colombia. One village belonged to the department César (Tezhumake) and three belonged to the department La Guajira (Ashintukwa, Siminke, and Cherua).

The study in 2020 was called “Colombia-Germany research program on diagnostics, research, treatment and prevention of Chagas disease and emerging infectious diseases in vulnerable groups”. Thereby, five villages were examined, all of which were located in the department César (Ahuyamal, Sabannah de Higuieron, Dungakare, Surimena), with the exemption of Potrerito, which belonged to the department La Guajira.

### 2.2. Screening

The screening procedure was identical across all villages. Individuals 12 years of age and older were at first tested with a Chagas rapid diagnostic test (RDT) from fresh blood specimens. If the test result was positive, further blood sampling was conducted. The blood serum was used to perform two different ELISAs, as required by a governmental directive, and a *T. cruzi*-specific real-time PCR assay called NDO-PCR was added. In volunteers below 12 years of age, serum was taken directly, as acute infections with CD are usually acquired in childhood. For all cases, RDT, ELISAs, and PCR were performed. The amount of blood taken from the children was adjusted according to the guidelines for pediatric patients [53]. The blood acquisition was accompanied by a complete physical examination performed by a physician; a questionnaire (medical history, Chagas-related signs and symptoms); an electrocardiogram (ECG); as well as measurements of vital signs, weight, and size.

Before enrolment into the drug observed treatment (DOT), a blood count and a laboratory–chemical analysis including standard liver enzymes and kidney parameters were conducted. In addition, a pregnancy test was performed for all women of childbearing age. Further, the assumed stage of CD was documented, and the pros and cons regarding CD-specific therapy were discussed with the patients. The majority of enrolled patients showed no signs or symptoms of a chronic CD, e.g., heart failure. Two cases with cardiac complaints were excluded: one case with NYHA IV cardiac dysfunction and one with a myocardial infarction. Others showed general ECG changes, e.g., AV I°, which could not be clearly attributed to CD, although this is considered a reasonable explanation. We elaborated on these findings in a separate paper [16].

### 2.3. Drug Observed Treatment

Drug observed treatment (DOT) groups were set up in each village equally. Patients with two positive ELISAs (as required by the Colombian guidelines) were included. Notably, RDT and ELISAs showed the same results in all instances, and the RDT was made additionally. If the patient was ELISA-positive and PCR-positive, he/she was, of course, also included. Patients with a negative serology and just a positive PCR were not found.

Each patient could participate if there were no contraindications (e.g., pregnancy, lactating stage, elevated liver values, etc.) and if the person agreed to allow blood acquisitions and to accept the behavioral requirements (in particular, safer sex) during therapy. For a treatment duration of two months, a physician was on site in the communities. The physician took care of the CD patients, but was also in charge of any other medical consultations reported by the Wiwas.

Benznidazole treatment was administered as follows: In adults, 5 mg/kg body weight, and in patients below 18 years, 10 mg/kg body weight were administered in 2 doses in the morning and night with food for 60 days (if no contraindications occurred under therapy).

Nifurtimox treatment is described as follows. For adults, 8–10 mg/kg body weight per day in 3 divided doses after meals was provided if no contraindications occurred. Children up to 10 years of age with acute infections orally received 15–20 mg/kg body weight per day in 3 divided doses after meals; for chronic infection, 15–20 mg/kg body weight per day in 3 divided doses after meals was orally administered. Children 1–16 years of age with acute infections took 12.5–15 mg/kg body weight per day in 3 divided doses after meals; for chronic infection, 12.5–15 mg/kg body weight per day in 3 divided doses after meals was orally applied. Children 17 years of age and older were treated like adults. All dosages were given for 60 days.

All patients, regardless of whether they were in the DOT program or not, received their diagnostic results. All positive CD cases were registered by the official health system and all positive patients received their entitlement for treatment, which was processed by health officials.

Medication was provided by the health authorities of Valledupar (Secretaria de Salud). To ensure compliance, medication was administered on site. Regular study check-ups were performed on study days 0, 7, 30, and 60. This included a questionnaire, anamnesis/medical history, and recording of side effects as well as signs and symptoms of CD. The intensity of the side effects were measured using a rating scale ranging from 1 to 5, with 1 expressing very low intensity and 5 expressing very high intensity. During all appointments, a full physical examination was conducted, including weight control and blood acquisition. Blood was taken to control possible side effects, in particular liver enzyme elevation, and to perform real-time PCR follow-up assessments over the course of the treatment. On day 60 (end of treatment), in addition, both ELISAs and the RDT were repeated. For all women of childbearing age, a weekly pregnancy test was performed. Patients were instructed to consult the doctor at any time in between regular visits if they required medical assistance and/or unusual symptoms/complaints occurred.

In total, 121 patients were treated with BNZ and 115 with NFX within the DOT. The provided medication, benznidazole (Abarax^®^) or nifurtimox (Lampit^®^), was adapted to the patient’s weight and administered following the manufacturer’s protocol for a duration of 60 days, as detailed above.

### 2.4. Serological Analysis

All samples were tested with a Chagas rapid diagnostic test (RT Chagas AB Rapid, Standard Diagnostics Inc. Bioline, Bogota, Colombia) and two ELISAs (Chagatest ELISA recombinante v. 4.0 Wiener Lab, Rosario, Argentina and Chagas IgG ELISA, IBL International GmbH, Hamburg, Germany), following the manufacturers’ protocols.

### 2.5. DNA Extraction

In 2018, DNA extractions from serum (200 µL) were eluted in 60 µL elution buffer following the instructions of the manufacturer’s protocol of the RTP Pathogen Kit (Invitek Molecular GmbH, “Ready-to-Prep”, Berlin, Germany) and stored thereafter at −20 °C. In 2020, extractions from serum (300 µL) were finally eluted in 80 µL elution buffer, following the instructions of the manufacturer of MagaBio plus Virus DNA/RNA purification kit version 2 (Hangszhou Bioer Technology Co., Ltd., Hangzhou, China). The extractions were performed in close temporal association to the blood acquisition events in order to avoid bias due to different states of DNA degradation within the samples. In a former assessment, it could be demonstrated that the use of the two different nucleic acid extraction kits did not have any relevant impact on the test results [54].

### 2.6. T. cruzi-Specific Real-Time-PCR (Newly Developed One-NDO)

The NDO-PCR was performed in 2018 as an in-house assay, as described previously [55]. In 2020, the kit of the assay was professionally produced by TibMolBiol, Berlin, Germany (*T. cruzi* Light Mix, Ref 53-0755-96, Phocid *Herpes Virus* (PhHV) Extraction control, reference 66-0901-96, Lyophilized 1-step RT-PCR polymerase mix, Cat.-No 90-9999-96) and used with the nucleic acid eluates as reported [56]. Real-time PCR was run on a Rotor-Gene Q cycler (Qiagen, Hilden, Germany) with reaction conditions exactly as detailed in the manual of the *T. cruzi* Light Mix assay, Ref 53-0755-96 (TibMolBiol). As a positive control, DNA from the *T. cruzi* strain Tulahuen (DTU TcII) was used in all PCR runs. As a negative control, PCR-grade water was used. All samples were assessed in duplicate. Further details on the PCR approach are provided in detail in the evaluation studies [54,55,56] and summarized below. In particular, the oligonucleotides used for the patent-protected NDO-PCR assay targeting kinetoplast minicircle DNA (GenBank accession number U07846.1) are provided in Table 1. Superior diagnostic accuracy as compared to other published or commercialized real-time PCR approaches was shown for the application with both hybridization probe variants [55,56], which are indicated in Table 1. In those evaluation assessments [55,56], excellent diagnostic accuracy values comprising sensitivity ranging between 92.3% and 97.9% as well as specificity ranging between 99.3% and 100% for the detection of *T. cruzi* DNA in human blood could be confirmed. This was associated with a very low detection threshold of only 1.5 target DNA copies per µL nucleic acid eluate, as assessed with a dilution series using DNA of the *T. cruzi* Tulahuen (DTU TcII) strain, as reported elsewhere [55]. Even more, NDO-PCR allowed for superior delineation of *T. cruzi* from non-pathogenic, but phylogenetically closely related, *T. rangeli* compared to competing in-house and commercial real-time PCR protocols in sequencing-controlled assessments [55,56].

### 2.7. Disease Classification

According to WHO classification [57], an acute phase or re-activated infection can be claimed if the parasite is detected using direct methods in the circulating blood. As real-time PCRs are direct methods, we classified a positive result with negative serology as an acute CD infection.

Chronic cases were divided in patients who were positive according to at least two serologic tests and negative according to all PCRs, as well as those positive according to at least two serologic tests and positive according to at least one PCR run. The last group was interpreted as re-infected, re-activated, and/or in an (early) chronic stage of a CD infection.

According to CDC definitions, the state of a prolonged asymptomatic form of CD during which only a few or no parasites are found in the blood stream can be classified as chronic indeterminate [58]. As the majority of patients in this study did not suffer from any complaints, this definition comprised the main share of the patients.

### 2.8. Data Management and Statistical Analysis

Statistical analysis was performed with the software R (version 4.2.3). Descriptive results were reported as mean (M), median (MD), standard deviation (SD), minimum (Min), and maximum (Max), if applicable. The Welch statistic was used for comparing two or more groups due to its documented superiority to other tests of significance with metric data [59,60,61]. Outliers were detected using 1.5× and 3 × 1.5 inter-quartile ranges (IQR), indicating normal and extreme outliers, respectively [62,63]. Chi-square testing was used in cases of nominal or ordinal data. Kernel density estimation was employed to visually display continuous development over time.

### 2.9. Ethical Clearance

The study in 2018 was approved by the Ethics Committee of Santa Marta, Colombia (Acta no. 032018). The study in 2020 was approved by the Institutional Ethic Committee for Investigation, Bogota, Colombia (Acta no. 2019-4). Written informed consent was obtained from each participant or from the parents or legal guardians of children prior to participation. The study was performed in accordance with the principles of the Declaration of Helsinki and all its amendments.

## 3. Results

The study population consisted of 944 persons, including 468 females. The mean age was 25.3 (±17.7) years; the oldest person was 90 years of age and the youngest only 1 year of age. Village (*Χ*^2^(8) = 14.35) (*p* = 0.073) and sex (*Χ*^2^(1) = 2.80) (*p* = 0.094) were not significantly associated with the prevalence of CD (cf. Table 1 for detailed demographic information). The included CD-positive individuals were chronically infected with CD, as indicated by two positive ELISAs (cf. Table 2). Notably, an association appeared for age: Individuals infected with CD were significantly older than individuals who tested negative (*t* (608.7) = −23.9) (*p* < 0.001). The population did not differ significantly between timelines regarding age (*t* (936.76) = −0.02) (*p* = 0.979), distribution of sex (*Χ*^2^(1) = 0.815) (*p* = 0.367) or persons testing Chagas-positive or -negative, respectively (*Χ*^2^(1) = 2.00) (*p* = 0.157).

**Figure 1:**
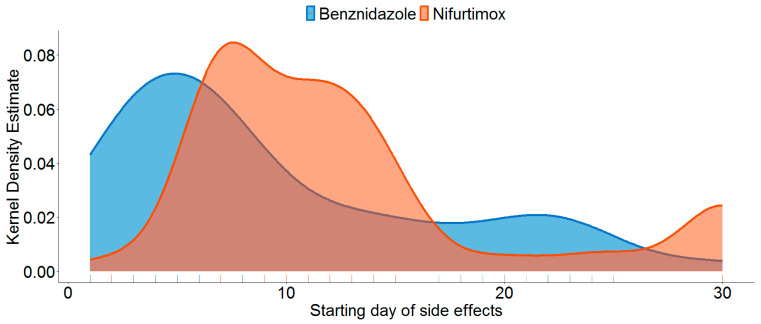
Beginning of complaints stratified by medication. Note. Kernel-smoothed prevalence of side effects showing the early onset of complaints for benznidazole (BZN) and the late onset for nifurtimox (NFX) with a reburst on day 30. Though treatment lasted sixty days, only one side effect appeared here (BZN). For this reason, this time period is not displayed.

In total, 220 patients tested Chagas-positive in 2018, when BZN was administered, and 160 in 2022, when NFX was provided. Of these, 121 patients could be treated with BNZ and 115 with NFX within the DOT. Overall, 115 individuals reported 209 side effects, of which 107 could be attributed to BZN and 102 to NFX. Thereby, BZN caused significantly fewer side effects (48.87%) than NFX (63.75%, *Χ*^2^(1) = 23.82) (*p* < 0.001). Patients continuously reported side effects from day 1 of receiving medication to day 60; however, the onset of complaints was significantly differently distributed (*Χ*^2^(35) = 102.98) (*p* < 0.001). For NFX, a late onset of side effects compared to BZN was recorded (cf. Figure 1). Unlike BZN, NFX had a peak of side effects on day 30 (cf. Table 3). Excluding extreme outlier values (duration > 10 days, which solely appeared for blood parameters), the average duration of side effects, measured in days differed highly significantly between BNZ (M = 1.53, SD = 1.37) and NFX (M = 2.39, SD = 1.70) (*p* < 0.001).

Using an intensity scale (ranging from 1 to 5), the side effects were rated significantly more severe for NFX (*M* = 2.11, *SD* = 0.51) than for BZN (*M* = 1.01, *SD* = 0.10) (*p* < 0.001). For two cases treated with NFX, the intensity of side effects was so severe that treatment had to be suspended. In detail, this was a 67-year-old man showing gastrointestinal complaints which could not be controlled with various medications. In the other case, a 48-year-old woman suffered from nausea and vomiting over 4 days. Associated with this, medication against these side effects lacked any therapeutic effect.

**Figure 2:**
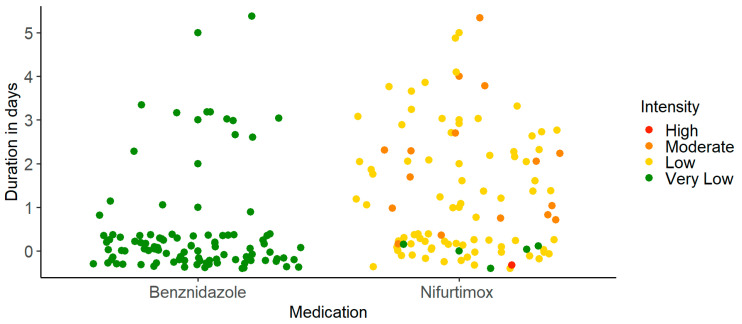
Duration in relation to intensity of recorded side effects. Note. Duration in days in relation to intensity of side effects displaying less severe as well as shorter side effects for BZN.

When assessing duration in relation to intensity, the burden of AEs caused by NFX was significantly higher compared to BZN (cf. Figure 2). The most common side effect for both medications was headache and dizziness, followed by gastrointestinal complaints. For BZN, skin rash and elevated blood parameters were more likely to occur.

When investigating the intensity of side effects within an individual over time, the intensity in case of NFX treatment increased ranging from 1.7 to 2.2, whereas the intensity in case of BNZ treatment stayed stable at 1.0. However, this tendency was not statistically significant.

Elevated blood parameters seen within the study primarily affected the liver enzymes, which were, in most cases, only marginally elevated or moderately increased (cf. Table 4). In all recorded cases, the elevated liver enzymes had decreased to a normal level in the subsequent control assessment, besides patient 4, for whom treatment was suspended earlier due to a severe Dengue infection (day 7).

**Figure 3:**
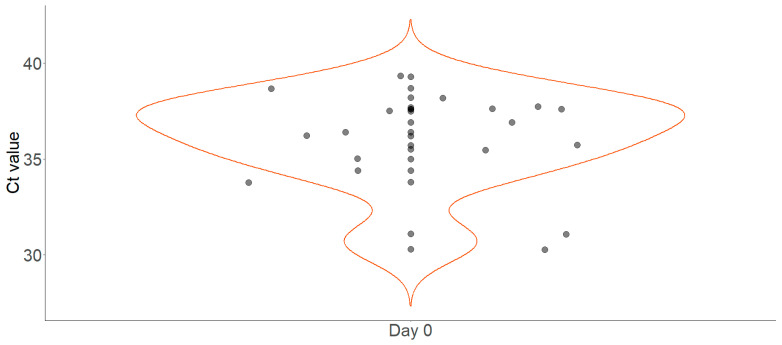
Violin plot displaying the distribution of measured *T. cruzi*-specific cycle threshold (Ct) values in real-time PCR. Note. Violin plot for day 0 depicting the distribution of Ct values by means of density estimation.

Favourable therapeutic outcomes were recorded for both study populations in terms of real-time PCR negativity. The mean cycle threshold (Ct) value of the positive tests on day 0 was 35.9 (2.5) (cf. Figure 3). Most of the patients were negative according to *T. cruzi*-specific real-time PCR on day 7 of therapy; only two patients were still positive on this day, with Ct values of 37.8 and 40.8, respectively. On day 30 of therapy, all patients tested negative. However, in one case, a negative PCR result turned positive on day 30 (Ct value = 37.2) under therapy. The next control showed a substantial decrease in the Ct value to 40.6 on day 60, and, subsequently, the treatment was nevertheless considered as successful. In a second case, a patient’s result turned positive during therapy on day 30 (Ct value = 37.2).

As one patient became pregnant shortly after the onset of treatment, her treatment was suspended and her outcome could not be tracked.

## 4. Discussion

Until today, there has been a controversial discussion regarding the question of which therapy for CD should be the first line: NFX or BNZ. This uncertainty can also be seen in case of the chosen study population, as the Colombian government changed their regimen between 2018 (BNZ) and 2020 (NFX). According to the Colombian Guidelines [64], in “terms of adverse effects, (….) it was determined that there are no substantial differences between the two drugs”. This finding could not be confirmed, in contrast. In a drug naïve population in the Sierra Nevada de Santa Marta, we examined relevant differences in a drug observed treatment comparing BNZ and NFX. The study population consisted of CD-positive patients, mainly without apparent clinical signs and symptoms or uncontrolled risk factors for cardiomyopathy. We were able to clearly demonstrate a superiority of BNZ as compared to NFX in the assessed indigenous population regarding observed side effects. Hereby, NFX’s AEs were more severe, lasted longer, and occurred more frequently.

The Colombia guideline also stated that “each drug has side effects, NFX more weight loss and psychiatric effects, BNZ more cutaneous and neurological reactions”. Although we found skin rashes to be more prominent in case of BNZ treatment, we did not record any specific neurological side effects. We can confirm that weight loss occurred more often in the NFX group; however, we did not find any psychiatric disorders. Instead, high numbers of gastrointestinal complaints were common in NFX-treated patients in our assessment. In two cases, gastrointestinal disorders even led to a withdrawal from CD therapy due to non-endurable symptoms. Furthermore, we found AEs like headache and dizziness to be the leading AEs, followed by gastrointestinal complaints, in both treatment arms.

Some authors claim that NFX might show a milder AE profile in children and adolescent patients compared to BNZ [23,35,40,41,43]. In our study, there were 24 patients between 7 and 18 years of age, of whom 16 were treated with BNZ and 8 with NFX. All participants stated complaints, but the intensity was rated lower in the BNZ group than in the NFX group. Furthermore, the duration of AEs was significantly longer for NFX (*M* = 2.39, *SD* = 1.70) compared to BZN (*M* = 1.53, *SD* = 1.37) (*p* < 0.001). This small but highly significant difference indicates a statistically robust effect [65]; thus, the aforementioned observation of milder NFX-associated AEs in children and adolescents could not be confirmed in our study. However, considering the AE rates in both study populations, our study confirms a need to develop new and more tolerable therapeutic options for Chagas disease. Previous reports on respective approaches have been summarized elsewhere [36,66,67,68,69,70,71,72,73,74,75,76,77,78,79,80,81,82,83,84,85,86,87], but as a summary, it can be stated that highly efficient new drugs still need to be developed.

In our study population, 48.9% stated at least one AE in the BNZ group and 63.8% in the NFX group. These numbers are low compared to other study results, e.g., as provided by Jackson and colleagues [18], where 84.8% versus 95.2% were reported. However, this might be due to the fact that the nociception of pain and complaints are different in the indigenous communities than elsewhere. This is understandable if one lives in a region where the next healthcare point is six hours away on foot. Next to this, we also served meals with the treatment with the intention of minimizing AEs. This could also be a reasons for the different results compared to the BENDITA trial performed in Bolivia [88], where fewer BNZ-associated side effects were recorded in the assessed indigenous population.

Notably, most AEs occurred in the BNZ treatment group around day 4 and day 7, and all of them could be coped with easily by the patients. In later stages of BNZ therapy, the number of AEs dropped significantly. In comparison, NFX-associated AEs showed various peaks, e.g., at days 7, 14, and 30. Also, a notable number of NFX-associated AEs occurred within the entire treatment period.

Most patients had already turned negative according to the very sensitive PCR after 7 days of treatment, while only two remained positive at this time point. In the PCR measurements on day 30, all results had become negative. Treatment failure may be postulated in the case of *T. cruzi*-specific PCR remaining positive. However, a successful cure cannot be definitively claimed just based on a negative PCR result, as, for example, the pathogen might persist just below the detection threshold. However, the study’s results nevertheless imply good anti-parasitic effects of both treatments, as a decline in parasitemia was undoubtedly seen. However, under NFX, either two newly gained infections or relapses were observed. A study investigating shorter treatment durations could nevertheless be promising, considering the results obtained with the highly sensitive real-time PCR approach used herein.

Focusing on the applied diagnostic strategy, direct parasite detection in the circulating blood was indeed performed with a very well-validated diagnostic real-time PCR assay targeting *T. cruzi* DNA in the human bloodstream. It had excellent diagnostic accuracy in terms of sensitivity and specificity, and was associated with a very low detection threshold and a superior discriminative power regarding the delineation of *T. cruzi* from apathogenic but phylogenetically closely related *Trypanosoma rangeli*, as detailed in the methods chapter above and in the evaluation studies [55,56]. In terms of the latter feature, the applied assay even outperformed an IVDR-(in-intro diagnostics regulation-) labeled commercial real-time PCR assay accredited for diagnostic use in line with the Regulation EU (European Union) 2017/746 in a recent sequencing-controlled evaluation study [56]. Comparable diagnostic accuracy of the PCR assay was confirmed for both applied nucleic acid extraction strategies in a previous validation assessment [53], which was performed to ensure reliable results irrespective of the chosen nucleic extraction protocol. Taking these facts together, we feel confident postulating that the applied molecular diagnostic strategy for the confirmation of active infection in terms of circulating pathogen DNA in the bloodstream comprises a well-evaluated, state-of-the-art technological approach providing very high diagnostic reliability.

Nevertheless, our assessment has a few limitations as well. The main limitation of the study is the fact that it could not be conducted as a double-blinded randomized controlled trial for funding reasons. For the same reason, long-term follow-ups for the assessment of relapses or reinfections could not be performed either. Future studies may investigate whether medication interacts with underlying (chronic) illnesses. The patients screened in this study had no apparent illnesses; however, elaborated diagnostic tests to validate these findings are of the utmost importance. As gastrointestinal infections are very common in the region, it cannot be ruled out that some side effects were related to new gastrointestinal infections instead of the medications. However, as we performed deworming before the study; as, e.g., liquid stool is taken as normal in the communities; and as the patients themselves claimed the adverse events to be directly related to the medication (e.g., by stating it appeared shortly after the intake and, e.g., disappeared after leaving the study), we feel justified in assuming that the majority of side effects were due to the treatment. If there was a bias, this occurred; however, both study arms showed a similar likelihood.

Finally, potential bias due to different assessment periods cannot be excluded, and more sampling would have been desirable as well, but was, unfortunately, unfeasible due to resource limitations of the study under the local conditions given. The study setting was affected by the COVID-19 pandemic in 2020, as described in more detail elsewhere [89,90], a situation which might have interfered with the registration of side effects.

## 5. Conclusions

In the indigenous study populations assessed herein, BNZ and NFX showed comparable therapeutic efficacy regarding the study’s surrogate parameter achievement of PCR negativity with a less severe AE profile for BNZ. The increased rate of therapy stops due to unbearable side effects as described for nifurtimox above was confirmed by our assessment. Altogether, the composition of observed side effects matched the expectations from previous reports [18,19,20,21,22,23,24,25,26,27,28,29,30,31,32,33,34,35,36,37,38,39,40,41,42,43,44,45,46,47,48,49,50]. Adding to experience from previous assessments, this study suggests that BNZ should be considered as a first-line drug for CD treatment. Further, the negative PCR results after 7 to 30 days might suggest that a treatment lasting 60 days might not be required, and studies to shorten the treatment duration should be performed; however, PCR negativity cannot provide definite proof of a clinical cure [37], as stated above.

## Figures and Tables

**Table 1:** Oligonucleotides used for the patent-protected *T. cruzi*-specific NDO-PCR assay.

Oligonucleotide Purpose	Oligonucleotide Name	Oligonucleotide Sequence	Reference
Forward primer	Chagas F	5′-GCACTATATTACACCAACCCC-3′	[55]
Reverse primer	Chagas R	5′-CATGCATCTCCCCCGTA-3′	[55]
Hybridization probe (as used for the original in-house assay)	Chagas S	5′-FAM-CGAACCCCACCTCC-BHQ1-3′	[55]
Hybridization probe (as used for the kit commercialized by TibMolBiol)	Chagas S2	5′-FAM-TCG+AACCCC+ACCTCC-BHQ1-3′	[56]

FAM = 6-Carboxyfluorescein, BHQ1 = Black Hole Quencher 1. The “+” symbol indicates locked nucleic acid (LNA) adenine bases included to alter the annealing temperature.

**Table 2:** Demographics stratified by village. CD-positive means positive according to at least two different ELISAs.

	*N* = 944	CD-Negative	CD-Positive	Age M (SD)	Sex (Female%)
Villages Overall		541 (57.3%)	403 (42.7%)	25.27 (17.7)	468 (49.6%)
Ahuyamal	90 (9.5%)	44 (48.9%)	46 (51.1%)	20 (15.6)	48 (53.3%)
Ashintukwa	107 (11.3%)	57 (53.3%)	50 (46.7%)	28 (22.3)	52 (48.6%)
Cherua	95 (10.1%)	51 (53.7%)	44 (46.3%)	25 (15.9)	49 (51.6%)
Dungakare	67 (7.1%)	45 (67.2%)	22 (32.8%)	26.3 (15.6)	32 (47.8%)
Sabannah de Higuieron	133 (14.1%)	77 (57.9%)	56 (42.1%)	24.9 (17.4)	71 (53.4%)
Potrerito	102 (10.8%)	72 (70.6%)	30 (29.4%)	31.7 (18.7)	50 (49%)
Seminke	111 (11.8%)	63 (56.8%)	48 (43.2%)	22.8 (15.5)	49 (44.1%)
Surimena	63 (6.7%)	34 (54%)	29 (46%)	22.3 (14.4)	32 (50.8%)
Tezhumake	176 (18.6%)	98 (55.7%)	78 (44.3%)	25.3 (18.1)	85 (48.3%)
sex (female)	468 (49.6%)	255 (54.4%)	213 (43.6%)	-	-
age *M* (*SD*)	25.27 (17.7)	15.46 (10.14)	38.44 (17.08)	-	-

Note. CD = Chagas disease, M = mean, SD = standard deviation.

**Table 3:** Characterization of side effects.

	Σ = 381	BZN (*n* = 121)	NFT (*n* = 115)	*p*
Side Effects (*N* = 209, 54.86%)		*n* = 107 (48.42%)	*n* = 102 (63.75%)	<0.001
Beginning of complaints (days, peaks (8 (%)))				<0.001
4		12 (11.22%)	0	
7		11 (10.28%)	33 (32.35%)	
14		3 (2.80%)	10 (9.80%)	
30		2 (1.87%)	11 (10.87%)	
Duration (days)				<0.001
0		82 (76.64%)	44 (43.14%)	
1		4 (3.74%)	15 (14.71%)	
2		1 (0.93%)	17 (16.67%)	
3		11 (10.28%)	12 (11.76%)	
longer than 3 days		7 (6.54%)	10 (9.80%)	
Intensity (on a scale 1–5)				<0.001
1 (=Very Low)		102 (95.33%)	7 (6.86%)	
2 (=Low)		1 (0.93%)	78 (76.47%)	
3 (=Moderate)		0	16 (15.67%)	
4 (=High)		0	1 (0.98%)	
5 (=Very High)		0	0	
Type of side effects				<0.001
Headache		53 (49.53%)	54 (52.94%)	
Dizziness		12 (11.21%)	21(20.58%)	
Gastrointestinal complaints		10 (9.35%)	17 (16.67%)	
Skin rash		11 (10.28%)	2 (1.96%)	
Elevated blood parameters		7 (6.54%)	0	
Weight loss		0	3 (2.94%)	
Nausea and vomiting		0	2 (1.96%)	
Muscular complaints		1 (0.93%)	1 (0.98%)	

**Table 4:** Measured elevated liver enzymes under therapy.

Patient.	GOT U/L	GOT U/L Ref. Range	GPT U/L	GPT U/L Ref. Range
1	74	0–50	84	0–65
2	75	<40	60	<41
3	161	<40	141	<41
4	167	<40	233	<41
5	65	<40	49	<41
6	124	<40	249	<41

Ref. range = reference range.

## Data Availability

All relevant data are provided in the manuscript. Raw data can be made available upon reasonable request.
